# Higher risk of recurrence in early-stage breast cancer patients with increased levels of ribosomal protein S6

**DOI:** 10.1038/s41598-024-75154-1

**Published:** 2024-10-24

**Authors:** Florencia Cascardo, Micaela Vivanco, María Cecilia Perrone, Andrea Werbach, Diego Enrico, Pablo Mando, Mora Amat, Paula Martínez-Vazquez, Javier Burruchaga, María Mac Donnell, Claudia Lanari, Ariel Zwenger, Federico Waisberg, Virginia Novaro

**Affiliations:** 1 Instituto de Biología y Medicina Experimental (IBYME) - Consejo Nacional de Investigaciones Científicas y Técnicas (CONICET), Buenos Aires, Argentina; 2 Instituto Alexander Fleming (IAF), Buenos Aires, Argentina; 3 Centro de Educación Médica e Investigaciones Clínicas (CEMIC), Buenos Aires, Argentina; 4 Hospital de Agudos “Magdalena V. de Martínez”, General Pacheco, Buenos Aires, Argentina; 5 Hospital Provincial de Neuquén “Dr. Castro Rendón”, Neuquén, Argentina; 6 Grupo Oncológico Cooperativo del Sur (GOCS), Neuquén, Argentina

**Keywords:** Breast cancer, Prognostic markers, PI3K/AKT/mTOR pathway, Predictive markers, Tumor relapse, Immunohistochemistry analysis, Cancer, Cell biology, Biomarkers, Oncology, Risk factors

## Abstract

PI3K/AKT/mTOR pathway is implicated in breast cancer progression and recurrence. The identification of *PIK3CA* and *AKT1* mutations and loss of PTEN serve as selection criterion for targeted therapies involving selective inhibitors. However, they do not consistently align with pathway activation, and high-cost determinations limit their routine application. PI3K-downstream epigenetic regulatory mechanisms broaden the alterations that amplify pathway activity and, consequently, sensitivity to selective inhibitors. In this retrospective observational study, conducted within a cohort of early-stage breast cancer patients, we determined phosphorylated ribosomal protein S6 (pS6) at Ser240/244 by immunohistochemistry as an indicator of PI3K pathway activation. Log-Rank test and Cox proportional hazards regression were used to analyze the clinical relevance of pS6, alone and together with clinicopathological variables, regarding recurrence-free survival. ROC curves and the area under the curves were used to evaluate the calibration and discrimination properties of uni- and multivariate models. Our results show that a high percentage of pS6 positive tumor cells was associated with an unfavorable prognosis in a cohort of 129 hormone receptor positive/HER2 negative breast cancer patients (Hazard Ratio = 5.92; Log-Rank *p* = 9.5e-08; median follow-up = 53 months). When assessed in combination with lymph node status, the predictive capacity was higher compared to both univariate models individually. In conclusion, pS6 could represent a novel independent marker for predicting recurrence risk in luminal breast cancer.

**Supplementary Information:**

The online version contains supplementary material available at 10.1038/s41598-024-75154-1.

## Introduction

Gene expression analyses have exerted a substantial influence over the past decade in breast cancer detection and management. Advanced molecular techniques, such as sequencing, have identified numerous tumor biomarkers with diagnostic, prognostic, and residual disease monitoring capabilities^1^. Additionally, classical molecular markers crucial in shaping the initial therapeutic approach for breast cancer include Ki67, estrogen receptor (ER), progesterone receptor (PR), and HER2. These biomarkers are conventionally assessed through immunohistochemistry, presenting itself as a cost-effective alternative for gene expression analyses in clinical practice, while retaining predictive and prognostic potential^1,2^.

In vitro, in vivo, and translational research have collectively demonstrated the pivotal role of the PI3K/AKT/mTOR pathway in diverse cellular processes, involving cell growth, proliferation, and differentiation. Furthermore, it stands, as well as TP53, as one of the most frequently mutated signaling pathways in solid tumors, including breast cancer where it is closely linked to therapy resistance and unfavorable prognosis^3,4^. Approximately 50% of breast tumors exhibit mutations within this signaling cascade or loss of the associated tumor suppressor PTEN. The prevalence of these alterations varies depending on the molecular subtype of the disease, as delineated by molecular status of ER and HER2^5^.

Recently, the identification of mutations in *PIK3CA* and *AKT1* genes, or loss of *PTEN*, detected in both tissue and circulating tumor DNA, has emerged as a resistance mechanism for disease progression after first-line therapy. These alterations led to the development of specific inhibitors against PI3Kα and pan AKT kinase, such as alpelisib and capivasertib, respectively, according to National Comprehensive Cancer Network (NCCN) guidelines^6^. Notably, treatment with either of these two inhibitors in combination with fulvestrant has shown to prolong progression-free survival against fulvestrant alone in patients with ER/PR positive and HER2 negative advanced breast cancer harboring *PIK3CA*- or *AKT1*-mutated or *PTEN* loss. The benefit was reported for patients who had previously undergone endocrine therapy, with or without a CDK4/6 inhibitor^7,8^.

However, it is essential to note that the presence of mutations in the *PIK3CA* or *AKT1* genes does not consistently correlate with heightened pathway activation^3,4^. The majority of *AKT* variants identified through tumor sequencing are characterized as silent mutations, with limited functional consequences^9^. Sakr and colleagues conducted a comprehensive analysis involving genotyping for hotspot mutations in *PIK3CA* and *AKT1*, along with immunohistochemical assessment of PTEN, INPP4B, phosphorylated AKT and S6 expression in breast cancer progression of ductal carcinoma in situ to invasive carcinoma^10^. Their findings revealed that activation of the PI3K pathway may occur through mechanisms beyond the loss of PTEN or INPP4B expression,  as well as mutations in *PIK3CA* or *AKT1*.

Considering the existing evidence, resistance to endocrine therapy involving the PI3K/AKT/mTOR pathway in ER positive/HER2 negative breast cancer remains not completely understood. Thus, we aimed to study the prognostic role of the downstream PI3K pathway effector pS6 in patients with ER positive/HER2 negative early-stage breast cancer.

## Results

Among our study cohort of 129 ER/PR positive/HER2 negative breast cancer patients (Table 1), where PI3K signaling alteration is expected to be prevalent^5^, we observed a median follow-up of 53 months (95% confidence interval (CI) of 50–71 months). At the time of diagnosis, pS6 showed significantly higher values in patients who experienced a recurrence event (*p* = 0.001), indicating an association of this phosphoprotein expression with disease relapse (Table 2). No discrepancies in pS6 levels were observed between luminal A-like and B-like subgroups (18.01% and 26.40%, respectively; *p* = 0.09) (data not shown). Additionally, clinicopathological parameters, including age (*p* = 0.04), tumor size (*p* = 0.003), lymph node status (*p* = 0.04), and stage (*p* = 0.001), were also associated with disease recurrence. Surprisingly, we did not find an association between Nottingham grade and disease recurrence in this group (*p* = 0.3).

**Table 1:** Clinicopathological characteristics of luminal breast cancer patients in the study cohort.

Characteristics		Values
n		129
Age (mean ± SD, years)		53.52 ± 11.75
Histologic subtype (n (%))	Ductal	95 (73.6)
	Lobular	18 (14.0)
	Ductal-lobular	12 (9.3)
	Others	4 (3.1)
Ki67 (median [IQR], %)		10.00 [5.00, 18.75]
Tumor size (median [IQR], cm)		3.00 [2.00, 5.50]
Lymph nodes (n (%))	Negative	44 (34.1)
	Positive	83 (64.3)
	Unknown	2 (1.6)
Stage (n (%))	I	22 (17.1)
	II	55 (42.6)
	III	52 (40.3)
Nottingham grade (n (%))	1	30 (23.3)
	2	63 (48.8)
	3	20 (15.5)
	Unknown	16 (12.4)
Therapy (n (%))	Hormonal	27 (20.9)
	Hormonal + chemotherapy	77 (59.7)
	Chemotherapy	8 (6.2)
	Unknown	17 (13.2)
Recurrence (n (%))	No	99 (76.7)
	Yes	30 (23.3)

Values are presented as n (%), mean ± SD or median [IQR], as appropriate. Disease recurrence was determined with a median follow-up time of 53 months (95% CI of 50–71 months). Other subtypes included papillary (*n* = 3) and micropapillary (*n* = 1) carcinomas. SD: standard deviation; IQR: interquartile range. Unknown refers to missing data.

**Table 2:** Clinicopathological characteristics of luminal breast cancer patients according to the occurrence of a recurrence event.

Characteristics		No recurrence (*n* = 99)	Recurrence (*n* = 30)	*p*-value
Age (mean ± SD, years)		54.69 ± 11.70	49.67 ± 11.25	0.04 *
Histologic subtype (n (%))	Ductal	71 (71.7)	24 (80.0)	0.4
	Lobular	13 (13.1)	5 (16.7)	
	Ductal-lobular	11 (11.1)	1 (3.3)	
	Others	4 (4.0)	0 (0.0)	
Ki67 (median [IQR], %)		11.00 [5.00, 20.00]	9.00 [3.25, 12.00]	0.1
Tumor size (median [IQR], cm)		2.60 [2.00, 5.00]	5.00 [2.58, 7.80]	0.003 *
Lymph nodes (n (%))	Negative	39 (39.4)	5 (16.7)	0.04 *
	Positive	59 (59.6)	24 (80.0)	
	Unknown	1 (1.0)	1 (3.3)	
Stage (n (%))	I	21 (21.2)	1 (3.3)	0.001 *
	II	47 (47.5)	8 (26.7)	
	III	31 (31.3)	21 (70.0)	
Nottingham grade (n (%))	1	27 (27.3)	3 (10.0)	0.3
	2	50 (50.5)	13 (43.3)	
	3	15 (15.2)	5 (16.7)	
	Unknown	7 (7.1)	9 (30.0)	
pS6 (median [IQR], %)		10.00 [1.00, 25.00]	45.00 [10.00, 67.50]	0.001 *

Values are presented as n (%), mean ± SD or median [IQR], as appropriate. Other subtypes included papillary (*n* = 3) and micropapillary (*n* = 1) carcinomas. *p < 0.05, using the Chi-squared test for categorical variables, and the one-way ANOVA test or Mann-Whitney U test for normal and non-normal continuous variables, respectively. Unknown data were excluded from the statistical analysis. SD: standard deviation; IQR: interquartile range.

**Fig. 1:**
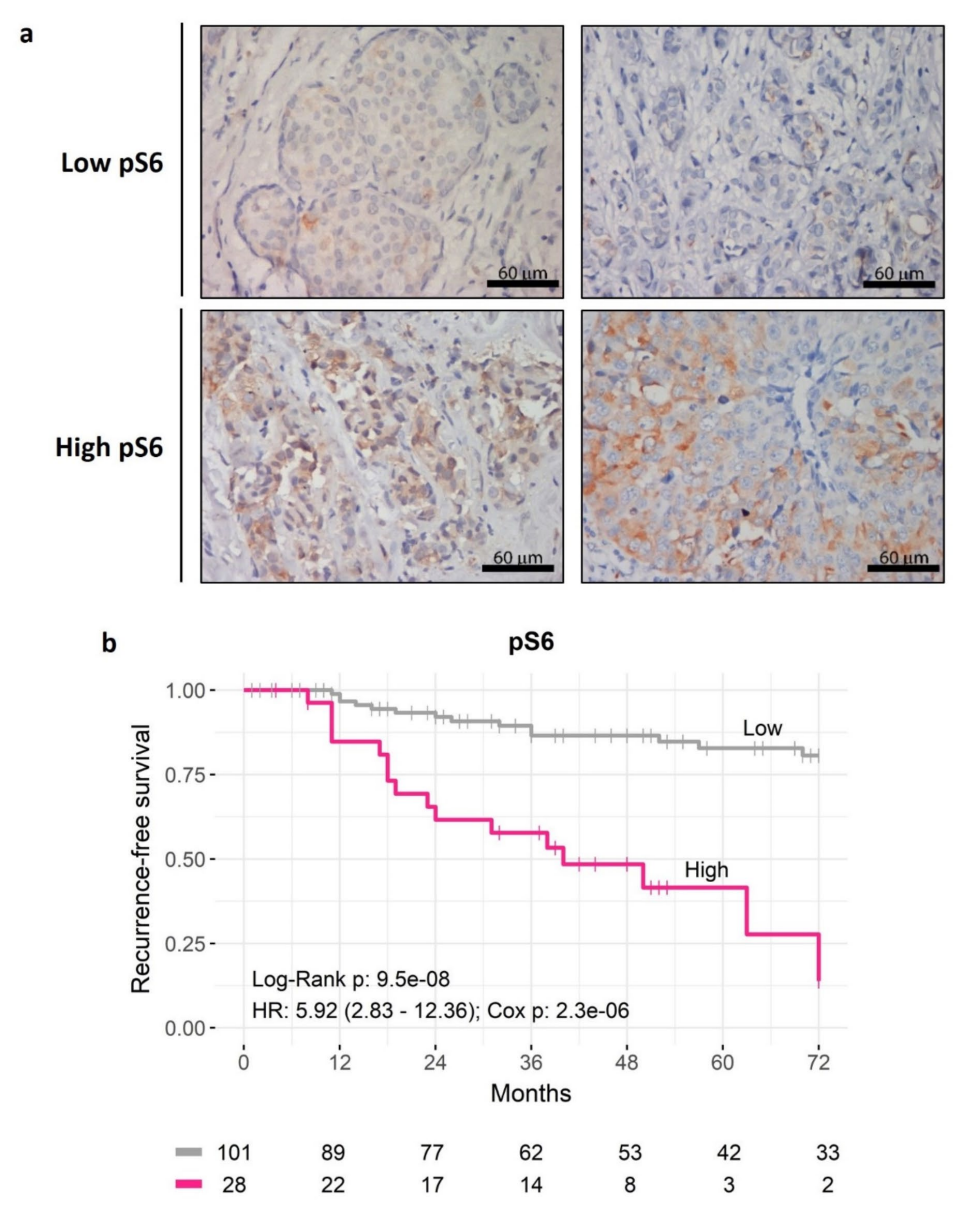
Analysis of pS6 as a risk predictor of recurrence in luminal breast cancer patients. (a) Immunohistochemistry images of tissues from four different patients, two representatives of low and two representatives of high pS6 level, determined by a cutoff of 42.5% pS6 positive tumor cells. Cytoplasmic perinuclear staining can be observed in all the samples. (b) Kaplan-Meier curves for recurrence-free survival (RFS) with low and high pS6 level. Log-Rank *p*, HR: hazard ratio (95% confidence interval), and Cox *p* are specified. Statistical significance was set at *p* < 0.05.

To further investigate this association of pS6 with disease recurrence, tumor tissue samples were categorized into two groups of low or high pS6 levels, after defining a cutoff of 42.5% pS6 positive tumor cells using the Log-Rank test-based method of the Cutoff Finder tool^11^ (Fig. 1a). Consequently, a univariate analysis showed that patients with high pS6 presented a significantly shorter recurrence-free survival (RFS) of 14.29% at 53 months compared to the 46.53% of those with low pS6 (HR = 5.92; Cox *p* = 2.3e-06; Log-Rank *p* = 9.5e-08) (Fig. 1b). It is worthwhile to mention that when we used the pS6 score instead of the percentage of pS6 positive tumor cells, we found similar results between low and high pS6 index (HR (95% CI) = 4.40 (2.09–9.28); Cox *p* = 9.9e-05; Log-Rank *p* = 2.2e-05).

**Fig. 2:**
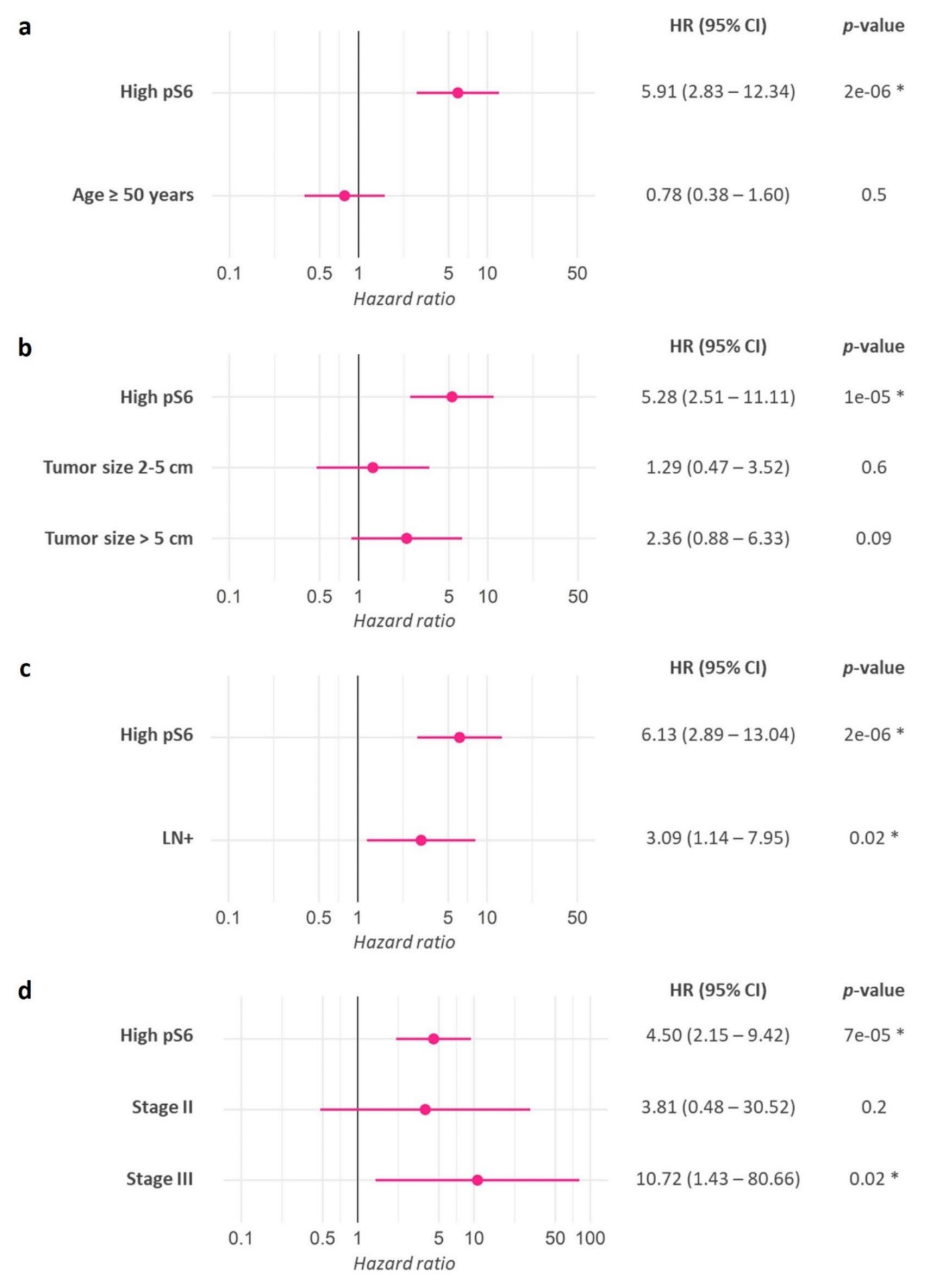
Multivariate analysis of pS6 and clinicopathological parameters as prognostic factors for RFS. Forest plots show the results obtained with pS6 and co-variables including (a) age (with less than 50 years as reference), (b) tumor size (with less than 2 cm as reference), (c) lymph node (LN) status (with negative LN as reference), and (d) stage (with stage I as reference). HR: hazard ratio; 95% CI: 95% confidence interval. Statistical significance was set at *p < 0.05.

Then, we examined the associations between pS6 levels and clinicopathological characteristics, finding that none of the variables related to recurrence identified in Table 2 (i.e. age, tumor size, lymph node status and stage) showed a significant dependence on pS6 expression (Table 3). Next, we selected these four parameters and included them with pS6 as co-variables in multivariate analyses (Fig. 2). When evaluated jointly within a model, high pS6 significantly increased the risk of recurrence (HR = 5.91; *p* = 2e-06) but age did not (Fig. 2a). Similarly, high pS6 incremented the risk of relapse (HR = 5.28; *p* = 1e-05) while tumor size did not (Fig. 2b). Both high pS6 (HR = 6.13; *p* = 2e-06) and positive lymph nodes (HR = 3.09; *p* = 0.02) showed statistical significance as powerful recurrence risk predictors by being assessed together (Fig. 2c). Finally, high pS6 (HR = 4.50; *p* = 7e-05) and stage III (HR = 10.72; *p* = 0.02) also represented increased risk of recurrence (Fig. 2d). Since Nottingham grade and therapy are validated prognostic factors, we also included them as co-variables with pS6 in multivariate analyses (Supplementary Fig. 1). In both cases, high pS6 significantly increased the risk of recurrence. Still, neither Nottingham grade (Supplementary Fig. 1a) nor therapy (Supplementary Fig. 1b) substantially contributed to the recurrence risk within the models analyzed.

**Table 3:** Clinicopathological characteristics of luminal breast cancer patients according to pS6 level.

Characteristics		Low pS6 (*n* = 101)	High pS6 (*n* = 28)	*p*-value
Age (mean ± SD, years)		53.54 ± 11.48	53.43 ± 12.92	0.9
Histologic subtype (n (%))	Ductal	69 (68.3)	26 (92.9)	0.07
	Lobular	17 (16.8)	1 (3.6)	
	Ductal-lobular	11 (10.9)	1 (3.6)	
	Others	4 (4.0)	0 (0.0)	
Ki67 (median [IQR], %)		9.00 [5.00, 17.75]	14.50 [4.75, 30.50]	0.2
Tumor size (median [IQR], cm)		3.00 [2.00, 5.00]	4.00 [2.50, 7.00]	0.06
Lymph nodes (n (%))	Negative	36 (35.6)	8 (28.6)	0.6
	Positive	63 (62.4)	20 (71.4)	
	Unknown	2 (2.0)	0 (0.0)	
Stage (n (%))	I	19 (18.8)	3 (10.7)	0.1
	II	46 (45.5)	9 (32.1)	
	III	36 (35.6)	16 (57.1)	
Nottingham grade (n (%))	1	28 (27.7)	2 (7.1)	0.04 *
	2	50 (49.5)	13 (46.4)	
	3	13 (12.9)	7 (25.0)	
	Unknown	10 (9.9)	6 (21.4)	
Recurrence (n (%))	No	87 (86.1)	12 (42.9)	< 0.001 *
	Yes	14 (13.9)	16 (57.1)	

Values are presented as n (%), mean ± SD or median [IQR], as appropriate. pS6 level represents the percentage of pS6 positive tumor cells, and the cutoff was determined as 42.5%. Recurrence was analyzed within a median follow-up time of 53 months. Other subtypes included papillary (*n* = 3) and micropapillary (*n* = 1) carcinomas. *p < 0.05, using the Chi-squared test for categorical variables, and the one-way ANOVA test or Mann-Whitney U test for normal and non-normal continuous variables, respectively. Unknown data were excluded from the statistical analysis. SD: standard deviation; IQR: interquartile range.

**Fig. 3:**
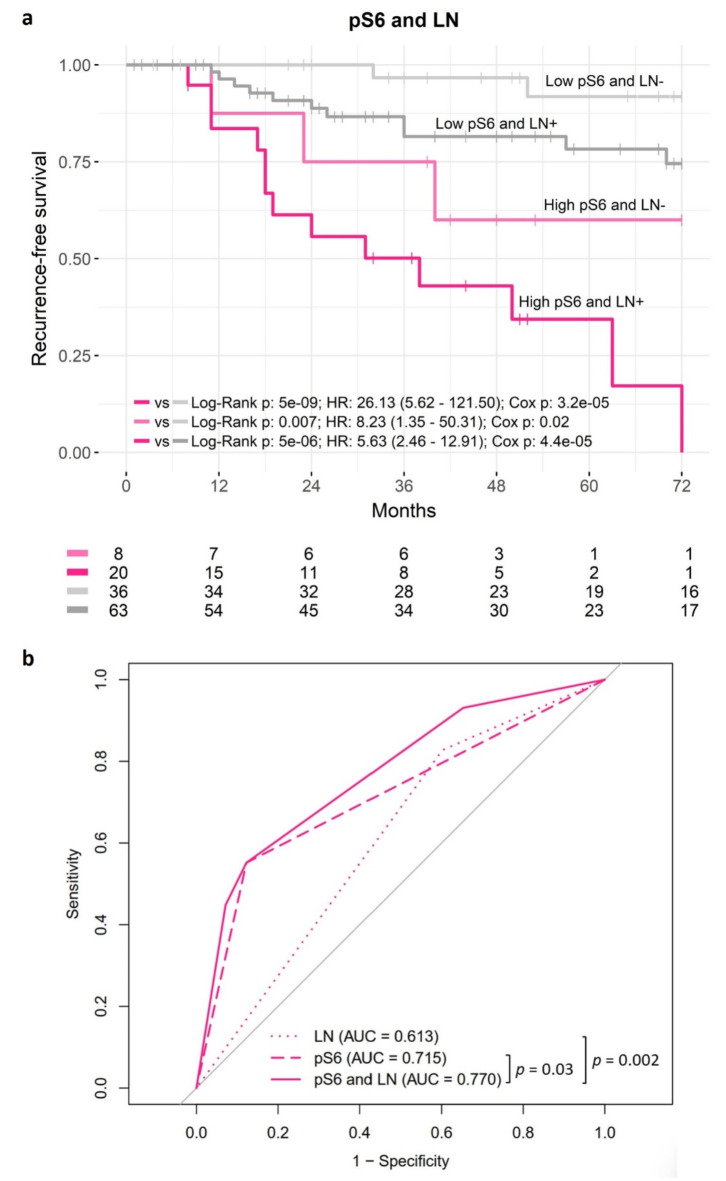
Analysis of pS6 and lymph node (LN) status as a combined risk predictor of recurrence in luminal breast cancer patients. (a) Kaplan-Meier curves for RFS with low and high pS6 jointly with negative and positive lymph nodes (LN- and LN+, respectively). Log-Rank *p*, HR: hazard ratio (95% confidence interval) and Cox *p* are specified. (b) ROC curves for LN and pS6, individually and combined. AUC: area under the curve. *p*-value using DeLong’s test. Statistical significance was set at *p* < 0.05.

The combined association of pS6 expression and lymph node status with RFS was analyzed in more depth, revealing that patients with the worst outcome were those with high pS6 and positive lymph nodes that had a RFS at 53 months of 10.00%, compared to patients with low pS6 and negative lymph nodes with a RFS of 52.78% (HR = 26.13; Cox *p* = 3.2e-05; Log-Rank *p* = 5e-09) or low pS6 and positive lymph nodes with a RFS of 44.44% (HR = 5.63; Cox *p* = 4.4e-05; Log-Rank *p* = 5e-06) (Fig. 3a). Moreover, patients with high pS6 and negative lymph nodes had a significantly shorter RFS of 25.00% compared to those with low pS6 and negative lymph nodes with a RFS of 52.78% (HR = 8.23; Cox *p* = 0.02; Log-Rank *p* = 0.007) (Fig. 3a).

An additional analysis to evaluate the discrimination and calibration properties of uni- and multivariate models incorporating pS6 as a biomarker in luminal breast cancer patients was performed by constructing a receiver operating characteristic (ROC) curve and calculating the area under the curve (AUC) as an indicator of the discriminatory capacity. Thus, although the predictive value of pS6 (AUC = 0.715) was not significantly higher compared to lymph node status (AUC = 0.613), the model that included both parameters combined increased the predictive capacity of each clinicopathological variable individually (AUC = 0.770; *p* = 0.002) (Fig. 3b).

## Discussion

This is the first study to propose that patients with early-stage luminal breast cancer who present higher tumor levels of pS6, as determined by immunohistochemical analysis, face a higher risk of relapse. Moreover, the prognostic value of pS6 appears to be even greater when combined with lymph node status. Ongoing validation studies are promising to incorporate pS6 as a novel and reliable independent indicator for risk stratification.

We present evidence suggesting that the evaluation of the level of phosphorylation of the ribosomal protein S6 at serine 240/244 (pS6 Ser240/244) through immunohistochemistry assays can be straightforwardly integrated into routine clinical practice as a surrogate for defining PI3K pathway activation. The specific findings in our study indicate that patients with tumors that display a higher percentage of pS6 positive cells had a higher risk of relapse. Considering the results presented in Figs. 2c and 3b, and that the risk of distant recurrence after 5 years of adjuvant endocrine therapy exhibited a strong correlation with the original node status and tumor grade^12^, analyzing pS6 levels provides further valuable prognostic information for luminal breast cancer. Consequently, we propose that immunohistochemical analysis of pS6 potentially could represent a valuable and easily implementable additional prognostic marker in clinical practice, particularly when assessed in combination with lymph node status.

Of note, other studies have linked the expression of components within the PI3K pathway to signaling activation. This is the case of the downstream effectors S6K1^13–16^ and pS6^17–19^, whose activation has been specifically associated with poorer prognosis. In contrast, a study by Ma and colleagues found that, despite the activation of mTOR and its downstream signaling components, including 4E‑BP1 and S6K1 in invasive breast cancer, these markers did not exhibit a statistically significant correlation with prognosis^20^.

Regarding S6K1, van der Hage and colleagues^16^ established it as an independent prognostic factor for locoregional recurrence predicting a poor rate in S6K1 overexpressing cases (HR (95% CI) = 2.67 (1.39–5.14); *p* = 0.003). Still, their study analyzed S6K1 by immunohistochemistry in a cohort of 452 node-negative premenopausal early-stage breast cancer patients with a median follow-up of 10.8 years. Discrepancies with our results in node-negative early-stage breast cancer patients with a median follow-up of 53 months (HR (95% CI) = 8.2 (1.35–50.31); *p* = 0.02) could be attributed to differences in the target protein analyzed or variations in the molecular subtype or menopausal status of the patients included in both studies.

In concordance with Cuperjani and colleagues^18^, our findings indicate that the expression of pS6 Ser240 is higher in HER2 positive breast carcinomas. While they reported a correlation between pS6 Ser240 expression and Ki67 index, we did not observe a similar correlation in our study (Table 3). This unexpected result may be attributed to variations in Ki67 analysis across different years and oncology centers in our study. Importantly, both in our study and in Cuperjani et al.^18^, patients with negative or lower pS6 Ser240 expression determined by immunohistochemical analysis exhibited significantly longer disease-free survival.

Existing literature suggests that in both primary and metastatic breast cancer, the majority of *PIK3CA* and *AKT1* mutations, or *PTEN* loss occurs predominantly in ER positive and HER2 negative cases, followed by HER2 positive cases, as opposed to basal-like tumors^5^. However, the association between pAKT1 Ser473 and phosphorylated S6K1 has been suggested as a marker indicating poor prognosis in HER2 positive tumors^21^. Moreover, *PIK3CA* mutations can impact the PI3K/AKT/mTOR signaling pathway independently of ER/PR and HER2 status. In this work, the analysis of the potential utility of pS6 as a prognostic marker was performed in the luminal subpopulation of patients, where PI3K signaling alteration is expected to be more critical and targeted therapies are more established.

While recently incorporated PI3K and AKT inhibitor-based therapies rely on droplet digital PCR (ddPCR) tests or next-generation sequencing (NGS) screening for *PIK3CA* and *AKT* hotspot mutations or *PTEN* loss, these FDA-approved tests are precise and convenient as they can utilize tumor tissue and/or circulating tumor DNA. However, their exclusivity and high cost currently impede their swift integration into routine clinical practice.

Moreover, only specific subsets of patients may derive benefits from PI3K/AKT/mTOR inhibitor-based therapy, particularly those with increased PI3K pathway activity^3,22^. Recent evidence indicates that the activation of the PI3K/AKT/mTOR signaling cascade in patients who develop progressive disease is driven by both genomic-dependent and independent mechanisms^23^. This involves several phosphorylated substrates, including pS6 and pAKT, among others^19^. Consequently, the significance of pS6 as a treatment response predictor may be underestimated when relying exclusively on genomic analyses as the source of molecular information.

Consistently with this observation, our research group has previously demonstrated in xenotransplants of endocrine-resistant cell lines and patient-derived tumor cells (PDCs) isolated from breast carcinomas that the effect of PI3K inhibitors is independent of the presence of *PIK3CA* mutations^24^. Furthermore, the overactivation of AKT and S6 through downstream mechanisms of PI3K does not necessarily involve point mutations^24^. Additionally, our research has illustrated that inhibiting tumor growth downstream at the mTOR/S6 level is more effective than inhibiting at the PI3K level when combined with endocrine agents and CDK4/6 inhibitors^24^. To further explore the potential of high pS6 levels as an indicator of an improved therapeutic response to PI3K, AKT or downstream inhibitors, additional preclinical and clinical studies are warranted to assess the predictive value of pS6.

Baseline activation of the PI3K/AKT/mTOR signaling axis may be linked to a lack of response to endocrine therapy when combined with CDK4/6 inhibitors^23,25^. Elevated expression of S6K1 in breast cancer has been proposed as a predictive marker for resistance to endocrine therapy^14,17^ or neoadjuvant chemotherapy^13^. In these scenarios, higher pS6 levels render breast cancer cells reliant on continuous pathway activation, analogous to an oncogene addiction. Conversely, low S6 phosphorylation during treatment is associated with a response to AZD5363/capivasertib in patient-derived xenografts from HER2 negative metastatic breast cancer^26^.

Exploring novel combinations of PI3K/AKT/mTOR inhibitors may offer a new perspective on breast cancer management, particularly for cases exhibiting resistance to endocrine therapy and a higher risk of recurrence. To this end, further studies are imperative to determine the clinical utility of pS6 as a downstream predictive marker for pathway inhibitors in the treatment of ER/PR positive breast cancer. The results that we present in this work suggest the need for an early “preventive” therapeutic strategy to mitigate the high risk of disease recurrence in cases with high pS6.

## Conclusion

In summary, phosphorylated S6 in Ser240/244 could represent a novel independent marker for predicting disease progression and recurrence risk in luminal breast cancer. It could also serve to identify patients who would benefit from PI3K/AKT/mTOR inhibitor-based therapy. Our findings provide new perspectives for enhanced individualized treatment guided by routine tumor analysis. Through this, we anticipate contributing to the improvement of survival outcomes for patients with high-risk breast cancer by offering an accessible tool for oncologists to make more selective and informed therapeutic decisions. This approach holds the potential to tailor treatments more precisely, leading to improved patient outcomes and overall advancements in breast cancer management.

### Limitations

The limitations of this study encompass its retrospective design, the relatively small sample size, and the imbalances observed in baseline patient characteristics and follow-up time. Additionally, unidentified confounding factors, including variations in the number of positive lymph nodes, in therapeutic regimens and oncology centers, may impact survival outcomes. Based on this preliminary data, we are conducting a larger study in a validation cohort to confirm the prognostic value of pS6 to identify patients with early-stage luminal breast cancer with higher risk of relapse.

Future investigations should involve a larger and more diverse cohort to enhance the generalizability of the findings and evaluate the predictive utility of pS6 in targeted therapies. Further research is needed also to evaluate the prognostic role of pS6 in patients undergoing adjuvant CDK4/6 inhibition in the actual setting for high-risk disease.

The inability to correlate the differential localization of pAKT Ser473 with clinicopathological parameters or disease-free survival in our study, with samples showing prevalent cytoplasmic labeling (HR = 2.63; Cox *p* = 0.2; *n* = 41) and others displaying prevalent nuclear labeling (HR = 1.29; Cox *p* = 0.8; *n* = 41), highlights the complexity of the PI3K/AKT/mTOR signaling pathway and the diverse mechanisms that contribute to its activation in cancer. Understanding the subcellular dynamics of AKT isoform components is crucial for unraveling the intricacies of signaling networks and their impact on tumor behavior^27^. Future studies with larger sample sizes and more detailed analyses of pAKT subcellular localization may provide additional insights into the clinical relevance of this molecular feature in breast cancer.

## Materials and methods

### Study design and patient selection

This retrospective cohort study included 129 patients diagnosed with stage I-III ER/PR positive/HER2 negative breast cancer treated in three Argentinian institutions: Instituto Alexander Fleming (IAF), Hospital de Agudos “Magdalena V de Martínez” (Pacheco), and Hospital Provincial de Neuquén “Dr. Castro Rendón” (HPN).

Female patients aged over 18 years old were included in our study, as long as they had available tumor samples and complete clinical data in their electronic medical records. Clinicopathological variables were assessed at the time of diagnosis. Main exclusion criteria encompassed prior diagnosis of other malignancies, hypertension or diabetes mellitus, and the identification of an advanced disease or pregnancy.

Data retrieved from hospital archives included patient age, tumor subtype, tumor size, lymphovascular invasion, lymph node metastasis, stage, Nottingham grade, and scheme of therapy. Recurrence was defined as any invasive breast cancer relapse, including locoregional relapse, distant metastasis, contralateral breast cancer, or primary malignancy in other organs. The study event of disease recurrence was evaluated with a maximum of a 6-year follow-up (72 months).

All specimens represent paraffin-embedded primary tumors and were acquired before any treatment, either as biopsies (IAF and HPN centers) or surgical specimens (Pacheco center) from 2009 to 2018.

Tumors were classified according to the St. Gallen Breast Cancer Guidelines 2021^28^. Luminal A-like tumors were defined considering positive ER expression, HER2 expression of 0, 1+, 2 + and non-amplified in situ hybridization (all of those defined as negative), PR cutoff equal or over 20% and Ki67 expression less than 20%. The remaining ER positive/HER2 negative and Ki67 more than 20% tumors were characterized as luminal B-like^2^.

### Immunohistochemistry (IHC)

Immunohistochemical (IHC) analysis was carried out manually at IBYME to determine tumor levels of pS6 and pAKT, as previously reported^24^. Briefly, microtome sections of 3–5 μm were obtained from tumors previously fixed in paraffin. The primary antibodies used were pAKT Ser473 (cat. #4060, Cell Signaling) and (cat. sc-33437, Santa Cruz Biotechnology), pS6 Ser240/244 (cat. #2215, Cell Signaling) and pS6 Ser235/236 (cat. #2211, Cell Signaling). The slides were subsequently exposed to biotinylated secondary antibodies (Vector Laboratories) for 1 h at room temperature, followed by a 30-min incubation with the Vectastain Elite ABC Kit (Vector Laboratories). Staining was carried out utilizing diaminobenzidine tetrahydrochloride solution (Vector Laboratories), followed by counterstaining with hematoxylin (Biopur, Santa Fe, Argentina), air-drying, and mounting with DPX (Sigma-Aldrich). Images were captured using a Nikon Eclipse E800 microscope equipped with the NIS-Elements software (Nikon Instruments Inc.).

Hematoxylin and eosin-stained tumor sections were evaluated by a staff hospital pathologist and a second pathologist, blinded to the clinicopathological data. The negative control of the IHC was established by eliminating the primary antibody. Staining intensity was categorized into no staining (0), low positive (+), positive (++), and high positive (+++) based on the intensity of the brown color observed in each view field. The score was calculated by multiplying the percentage of positive cells with the intensity of the staining. Since the scoring data displayed more dispersion and seemed to be operator-dependent, we opted to utilize the percentage of positive tumor cells in most of the tables and figures. The mean percentage and score of pS6 and pAKT positive cells determined by two observers were employed for statistical analyses.

Each preparation was examined using a light microscope at 200x and 400x magnification. The percentage of pS6 and pAKT positive cells were evaluated in five high-power visual fields (400x) selected randomly; each region was represented by nearly 100 tumor cells. pS6 positivity was indicated by cytoplasmic perinuclear brown staining of the tumor cells, while pAKT positivity was indicated by nuclear or cytoplasmic brown staining, similar as reported^18,27^.

Despite evaluating phosphorylated S6 at both Ser235/236 and Ser240/244 in nearly 50% of the tissues and obtaining similar results, we chose to focus on the latter. This decision is based on prior evidence suggesting that S6K1 and S6K2 primarily phosphorylate Ser240 and Ser244, while they play a dispensable role in Ser235 and Ser236 phosphorylation^29^. Furthermore, it has been demonstrated that Ser235/236 requires extracellular signal-regulated kinase (ERK) signaling in an mTOR-independent mechanism that is unresponsive to rapamycin^30^. In conclusion, the expression of pS6 residues 240/244 emerged as a more precise marker of mTORC1 signaling.

### Statistical analysis

Patients were divided into groups of low and high percentage of pS6 positive tumor cells, establishing a cutoff value with the minimal Log-Rank *p* method from the Cutoff Finder tool^11^. The study events and disease recurrence were evaluated at a maximum of a 6-year follow-up (72 months). Subgroup analysis, associations and confounding factors were evaluated, including key patient characteristics, such as tumor and lymph node staging, Ki67, and Nottingham grade. Associations between recurrence or pS6 levels and the rest of the clinicopathological parameters were evaluated using the Chi-squared test for categorical variables, and the one-way ANOVA test or Mann-Whitney U test for normal and non-normal continuous variables, respectively. To determine the effect of the variables of interest on patients’ RFS, Kaplan-Meier curves were constructed, and the Log-Rank test and Cox proportional hazards regression were used complying with the rule of thumb which states that at least 10 events per candidate predictor are required to obtain reliable results. Receiver Operating Characteristics (ROC) curves and the area under the curve (AUC) were implemented to analyze the performance of pS6 and lymph node status, individually and combined, and statistically significant differences were identified using DeLong’s test.

Statistical significance was set as *p* < 0.05. The analyses were performed using R Statistical Software 4.3.2 (R Core Team, 2023), RStudio software (Posit Software, PBC, Boston, MA, USA), and dplyr (v1.1.3), survival (v3.5-7), ggplot2 (v3.4.4), survminer (v0.4.9), survivalAnalysis (v0.3.0), tableone (v0.13.2), sjPlot (v2.8.15) and pROC (v1.18.5) packages.

## Electronic supplementary material

Below is the link to the electronic supplementary material.

Supplementary Material 1

## Data Availability

The authors declare that anonymized data supporting the results can be accessed upon request to the corresponding author VN.
