# Supplementary material for: Higher risk of recurrence in early-stage breast cancer patients with increased levels of ribosomal protein S6
Source: Sci Rep. 2024 Oct 24;14:25136. doi: 10.1038/s41598-024-75154-1 (PMC11502685; doi:10.1038/s41598-024-75154-1)
Supplement: Supplementary file 1 — Supplementary Material 1 [file 41598_2024_75154_MOESM1_ESM.pdf]

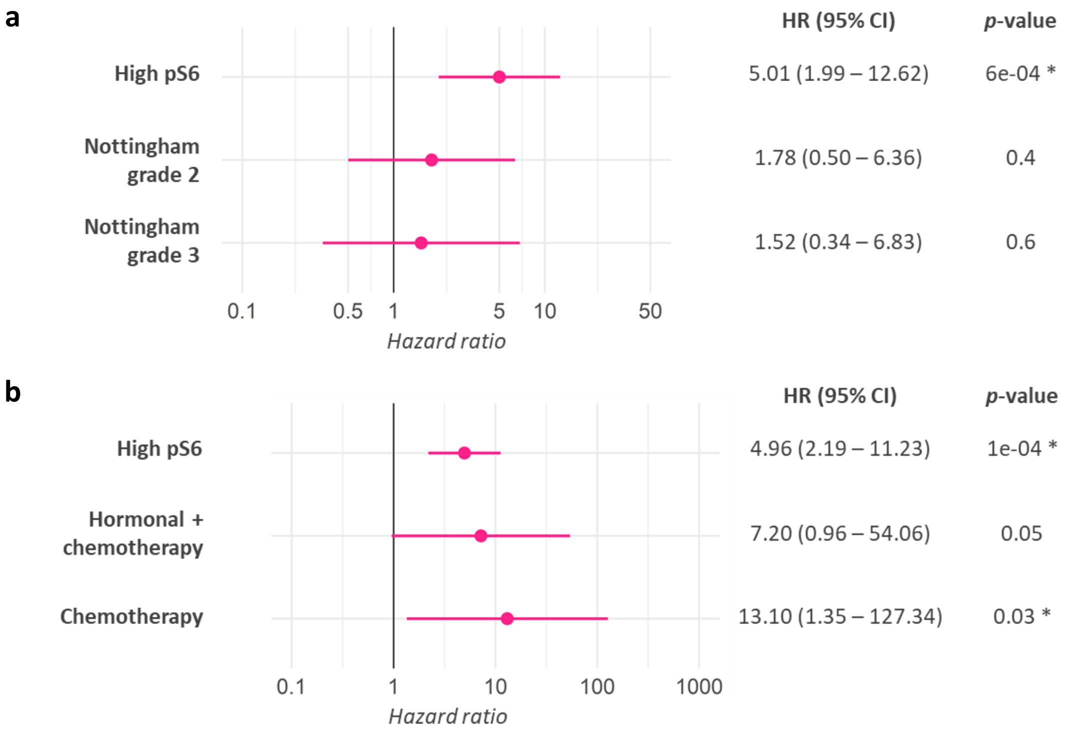

Supplementary Figure 1. Multivariate analysis of pS6 and clinicopathological parameters as prognostic factors for RFS. Forest plots show the results obtained with pS6 and co-variables including **a.** Nottingham grade (with grade 1 as reference) and **b.** therapy (with hormonal therapy alone as reference). HR: hazard ratio; 95% CI: 95% confidence interval. Statistical significance was set at *$p < 0.05$.
